# A meta-analysis of Watson for Oncology in clinical application

**DOI:** 10.1038/s41598-021-84973-5

**Published:** 2021-03-11

**Authors:** Zhou Jie, Zeng Zhiying, Li Li

**Affiliations:** 1 Department of Gynecologic Oncology, Guangxi Medical University Cancer Hospital, Key Laboratory of Early Prevention and Treatment for Regional High Frequency Tumor, Ministry of Education, Nanning, 530021 Guangxi People’s Republic of China; 2 Department of Gynecology, The Second Affiliated Hospital, University of South China, Hengyang, 421001 Hunan People’s Republic of China; 3 Department of Anesthesiology, The Second Affiliated Hospital, University of South China, Hengyang, 421001 Hunan People’s Republic of China

**Keywords:** Medical research, Oncology

## Abstract

Using the method of meta-analysis to systematically evaluate the consistency of treatment schemes between Watson for Oncology (WFO) and Multidisciplinary Team (MDT), and to provide references for the practical application of artificial intelligence clinical decision-support system in cancer treatment. We systematically searched articles about the clinical applications of Watson for Oncology in the databases and conducted meta-analysis using RevMan 5.3 software. A total of 9 studies were identified, including 2463 patients. When the MDT is consistent with WFO at the ‘Recommended’ or the ‘For consideration’ level, the overall concordance rate is 81.52%. Among them, breast cancer was the highest and gastric cancer was the lowest. The concordance rate in stage I–III cancer is higher than that in stage IV, but the result of lung cancer is opposite (*P* < 0.05).Similar results were obtained when MDT was only consistent with WFO at the "recommended" level. Moreover, the consistency of estrogen and progesterone receptor negative breast cancer patients, colorectal cancer patients under 70 years old or ECOG 0, and small cell lung cancer patients is higher than that of estrogen and progesterone positive breast cancer patients, colorectal cancer patients over 70 years old or ECOG 1–2, and non-small cell lung cancer patients, with statistical significance (*P* < 0.05). Treatment recommendations made by WFO and MDT were highly concordant for cancer cases examined, but this system still needs further improvement. Owing to relatively small sample size of the included studies, more well-designed, and large sample size studies are still needed.

## Introduction

With the rapid development of human society, cancer-related knowledge is also growing exponentially, which has caused a knowledge gap for clinic physicians^1^. With the increasing understanding of each patient, more and more information need to be absorbed from the literature in providing evidence-based cancer treatment. Research shows that clinic physicians can only spend 4.6 h a week to acquire the latest professional knowledge^2^, resulting in a relative delay in information absorption, leading to an increasing gap between the results achieved by academic research centers and the actual situation^3^. However, compared with physicians in other clinical disciplines, clinical oncologists urgently need to acquire evidence-based medicine knowledge in time to support patients' personalized treatment plans. Consequently, clinicians need some new types of tools to bridge this knowledge gap, support and adopt new treatment methods in an evidence-based manner, so that more patients can benefit from social investment in research and development^4,5^. Artificial intelligence (AI) first appeared in the early 1950s, which refers to the creation of intelligent machines with functions and reactions like human beings^6^. The goal of AI is to replicate human mind, that is to say, it can perform tasks such as identification, interpretation, reasoning and transformation, and it is good at the areas that human beings are not good at, such as absorbing a large amount of qualitative information that can recognize the patterns of relevant information^7,8^. Now AI has gradually entered medicine. Image recognition using AI has been successfully applied to image-based clinical diagnosis, such as melanoma recognition in dermoscopy images^9^ or detection of diabetic retinopathy in retinal fundus photographs^10^, and more and more researches on AI are also carried out in oncology^11–14^. AI aims to enhance human capabilities, enable human beings to apply more and more complex knowledge to clinical decision-making, and bring more and more diversified and complex patient data into personalized management. Due to the recent development of cognitive computing technology, its application in clinical oncology still lacks large-scale data, and there are clinical differences in different regions and ethnic groups. Watson for Oncology (WFO), an artificial intelligence assistant decision system, was developed by IBM Corporation (USA) with the help of top oncologists from Memorial Sloan Kettering Cancer Center (MSK). It took more than 4 years of training, based on national comprehensive cancer network (NCCN) cancer treatment guidelines and more than 100 years of clinical cancer treatment experience in the United States, and can recommend appropriate chemotherapy regimens for specific cancer patients. As for supported cases, the treatment recommendations provided by WFO are divided into 3 groups: Recommended, i.e. green "buckets", which represents a treatment supported by obvious evidence; For consideration, i.e. yellow "buckets", which represents a potentially suitable alternative; and Not recommended, i.e. red "buckets", which stands for a treatment with contraindications or obvious evidence against its use. In order to compare the consistency between WFO and clinicians in different countries and regions in various aspects and on a large scale, many hospitals have formed Multidisciplinary Team (MDT), which is composed of oncologists, surgeons, pathologists and radiologists, etc. They discuss the advantages and disadvantages of each candidate treatment scheme and finally determine the treatment scheme. If the concordance is achieved when the MDT recommendation is in the ‘Recommended’/‘Recommended’ or ‘For consideration’ categories of WFO, it is defined as concordant; Otherwise, it is discordant. The results showed that there were obvious differences in the concordance rate of different regions and types of cancers. And so far, there has been no published meta-analysis comparing the consistency of WFO and MDT. Therefore, this study aims to systematically review the literature and provide the latest evidence of WFO's clinical use, analyze the consistency, advantages and disadvantages between WFO's treatment scheme in cancer patients and that of clinicians, and further summarize and analyze WFO's clinical practice, so as to provide references for further clinical application of WFO.

## Materials and methods

This meta-analysis is registered in the International Prospective Register of Systematic Reviews (PROSPERO) trial registry (CRD42020199418). In addition and where applicable, the general guidelines of the Preferred Reporting Items for Systematic Reviews and Meta-Analysis (PRISMA) Statement were followed. And this study was performed and prepared according to the guidelines proposed by Cochrane Collaboration (http://www.cochrane-handbook.org).

### Literature search

Since WFO started commercial use in 2015, literatures from 2015 onwards were searched. Cochrane Library, PubMed, Excerpta Medica Database (EMbase), China National Knowledge Infrastructure (CNKI), CQVIP and Chinese Biomedicine (CBM) databases (updated until December 31, 2019) were searched using the following terms: artificial intelligence, clinical decision-support system, Watson for Oncology, neoplasm, treatment, Multidisciplinary Team, concordance and comparative study. Other potentially qualified articles were also screened manually.

### Inclusion and exclusion criteria

The studies meeting the following criteria would be included:

(a) The clinical use of WFO has been focused on regardless of cancer type, (b) the studies contain at least one subgroup of analysis data, (c) the studies should be original research articles published either in Chinese or English regardless of nationality, (d) the studies have compared the consistency of treatment schemes determined by WFO and MDT, and (e) there is no limit to whether the article is a prospective or a retrospective study and whether blind methods have used.

The following are the major exclusion criteria:

(a) The studies only describe the simple use of WFO and do not involve any data or only WFO research and development process data, (b) the article does not compare the treatment schemes between WFO and MDT, and (c) book chapter, comment, case reports, and other forms without detailed data.

### Data extraction and quality assessment

Two investigators evaluated the quality of the literatures and extracted the data independently. Any disagreements were discussed and consulted by an additional independent arbitrator for further resolution. The lack of original data is supplemented by contacting the original author via e-mail. The data were extracted with a standardized table, including (a) general information, such as the title of the publication, first author’s surname, the original document number and source, year of publication and country, (b) research characteristics, such as the eligibility of the research, the characteristics of the research object, the design scheme and quality of the literature, the design scheme and quality of the literature, the specific contents and implementation methods of the research measures, relevant bias prevention measures, and the main test results; (c) data needed for this meta-analysis, such as the total number of cases in each group, and the number of cases of events were collected by the second classification.

According to the Cochrane Reviewers’ Handbook 6.1 (http://www.cochrane-handbook.org), the quality of the literature was evaluated including 7 aspects: random sequence generation (selection bias), allocation concealment (selection bias), blinding of participants and personnel (performance bias), blinding of outcome assessment (detection bias), incomplete outcome data (attrition bias), selective reporting (reporting bias) and other bias, and the judgment of "yes" (low bias), "no" (high bias) and "unclear" (lack of relevant information or uncertainty of bias) is made respectively. Review Manager statistical software (RevMan, version 5.3.5, Cochrane Collaboration Network) was applied to assess the risk-of-bias and provide visual results.

### Statistical analysis

RevMan 5.3.5 was also applied to analyze the extracted data. The main purpose of this study was to compare the consistency of treatment schemes determined by WFO and MDT in different cancer types, so the statistical data were dichotomous data (coincidence or non-coincidence). In the analysis, odds ratios (ORs) and the 95% confidence intervals (CIs) were performed for clinic-pathological features (TNM stage, histopathological category, etc.). Q test or I^2^ test was used to judge the heterogeneity among the studies. When *P* < 0.05 or I^2^ > 50%, there was significant heterogeneity among the studies. On the contrary, there was no heterogeneity. When there was no statistical heterogeneity between studies, the fixed effect model was used to merge the results. If there was statistical heterogeneity, we analyzed the causes of heterogeneity, and adopted subgroup analysis or sensitivity analysis. For the documents that still could not eliminate heterogeneity, the data could be combined from the perspective of clinical significance. Random effect model was adopted for combination analysis, and the results were carefully interpreted. If the data provided could not be meta-analyzed, only descriptive analysis would be done.

## Results

### Characteristics and quality evaluation of eligible studies

**Figure 1:**
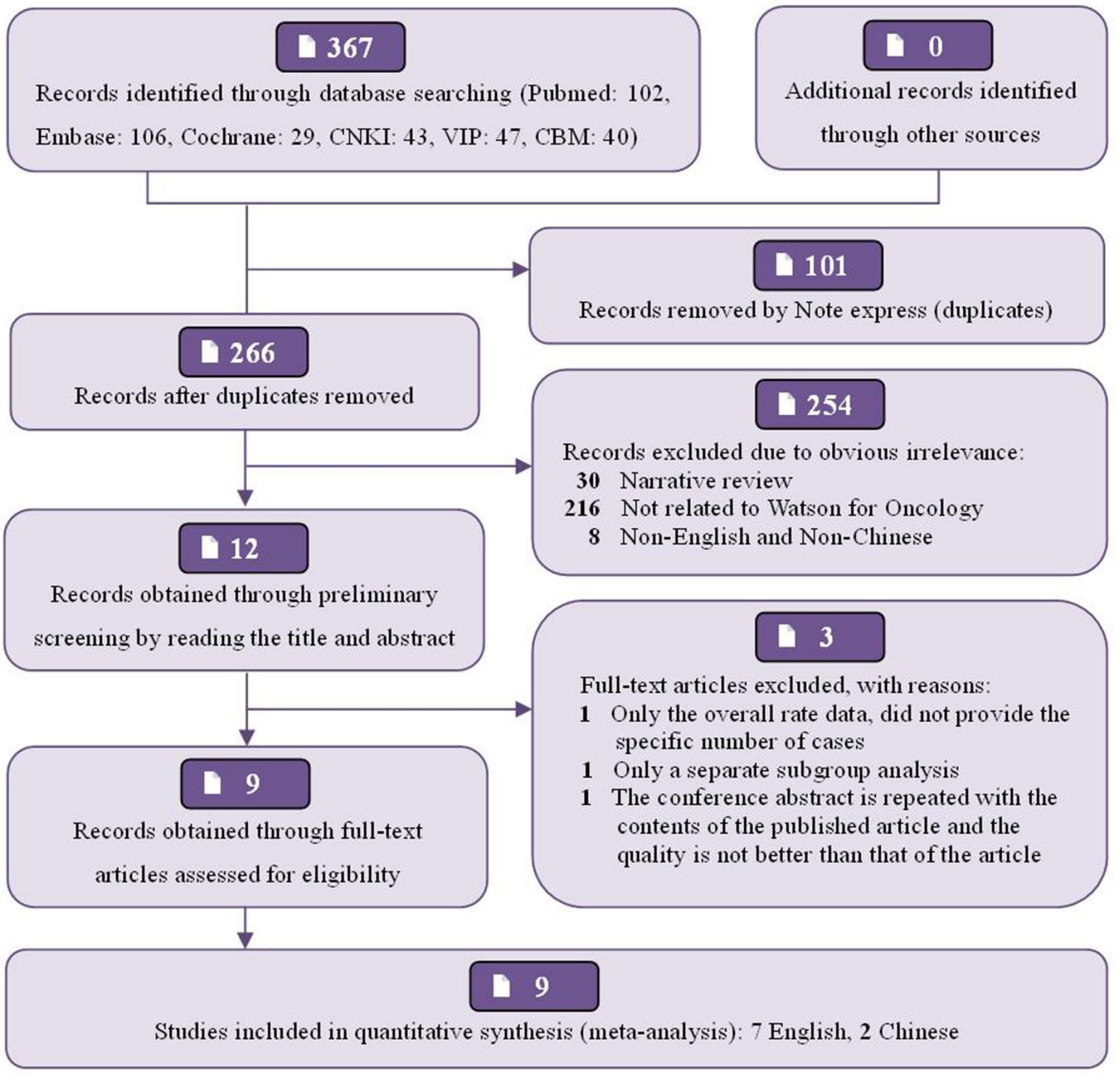
Flow diagram of the study selection process.

A total of 367 relevant publications from January 2015 to December 2019, were obtained from the preliminary search. There were 237 English literatures (Pubmed: 102, Embase: 106, Cochrane Library: 29) and 130 Chinese literatures (CNKI: 43, CQVIP: 47, CBM: 40). After reading the title, abstract and full text successively, 8 articles^15–22^ and 1 conference abstract ^23^ were finally included, all of which were Non-RCTs published between 2017 and 2019, 7 studies^15–17,19,20,22,23^ were published in English, and 2 studies^18,21^ in Chinese. The basic process of publication selection, the main characteristics and quality evaluation of included publications have been shown in Fig. 1, Table 1, Supplementary Fig. 1, 2, respectively. Of the 9 studies, 7 studies^15–17,19–22^ clearly defined the method of selecting cases, and other studies did not indicate the "randomization" of the included samples. In all studies, WFO and MDT treatment schemes were formulated successively for the same patient in the group, so there was no allocation bias. 7 studies^15,16,18–22^ did not indicate specific blind method implementation plan or did not adopt blind method, but the result judgment and measurement will not be affected. Although two studies^16,22^ did not provide detailed four-category data, they did not completely affect our meta-analysis, so we believed that all studies had no obvious bias in selective reporting results and ensured the basic integrity of the data, but other biases were still unclear. Because it was of little significance to use Begg’s funnel plot and Egger test to detect publication bias when the number of documents was too small (< 10), no publication bias analysis had been performed in this study. Due to the little difference in the quality of the documents included in this meta-analysis, no further sensitivity analysis had been made. After subgroup analysis, most I^2^ test results were less than 50%, and there was lower heterogeneity among the studies included in this system evaluation.

**Table 1:** Main characteristics of publications included in this meta-analysis.

Study	Languages	Type	Country	Sample size	Cancer type	Details of WFO treatment schemes	Outcome measure
Choi 2019^15^	English	Article	Korea	65	Gastric cancer	R + C + NR + NA	①
Kim 2019^16^	English	Article	Korea	69	Colorectal cancer	R + C + (NR + NA)^a^	①③④
Zhou 2019^17^	English	Article	China	362	Colon, rectal, lung, breast, stomach, cervical and ovarian cancer	R + C + NR + NA	①②
Hu 2018^18^	Chinese	Article	China	30	Colon cancer	R + C + NR + NA	①
Liu 2018^19^	English	Article	China	149	Lung cancer	R + C + NR + NA	①②
Somashekhar 2018^20^	English	Article	India	638	Breast cancer	R + C + NR + NA	①②
Xu 2018^21^	Chinese	Article	China	132	Breast cancer	R + C + NR + NA	①②
Lee 2018^22^	English	Article	Korea	656	Colon cancer	R + (C + NR + NA)^a^	①③④
Somashekhar 2017^23^	English	Conference abstract	India	362	Colon, rectal and lung cancer	R + C + NR + NA	①

*R* Recommended, *C *for consideration, *NR *not recommended, *NA *not available.

① Clinical staging, ② Pathological subtypes, ③ ECOG performance status, ④ Age.

^a^The data in brackets represent that only the merged data were given, and not each item of data was given separately.

### Results of meta-analysis

#### Overall analysis of consistency between WFO and MDT

Of the 9 included studies, a total of 7 studies^15,17–21,23^ provided four types of complete data (including WFO three types of treatment schemes and unavailable cases) on the consistency of treatment schemes determined by WFO and MDT in different cancer types, involving seven types of cancers including breast cancer, rectal cancer, colon cancer, gastric cancer, lung cancer, ovarian cancer and cervical cancer. Of the 1738 cases included (shown in Supplementary Fig. 3), 959 (55.18%) cases were WFO ‘Recommended’ schemes (green schemes) that were consistent with MDT treatment schemes, 503 cases (28.94%) were ‘For consideration’ (orange schemes), and the sum of the two was 1462 cases (84.12%). However, there were 166 cases (9.55%) that were ‘Not recommend’ scheme (pink scheme) and 110 cases (6.33%) that were not supported by WFO (‘Not available’ scheme).

**Figure 2:**
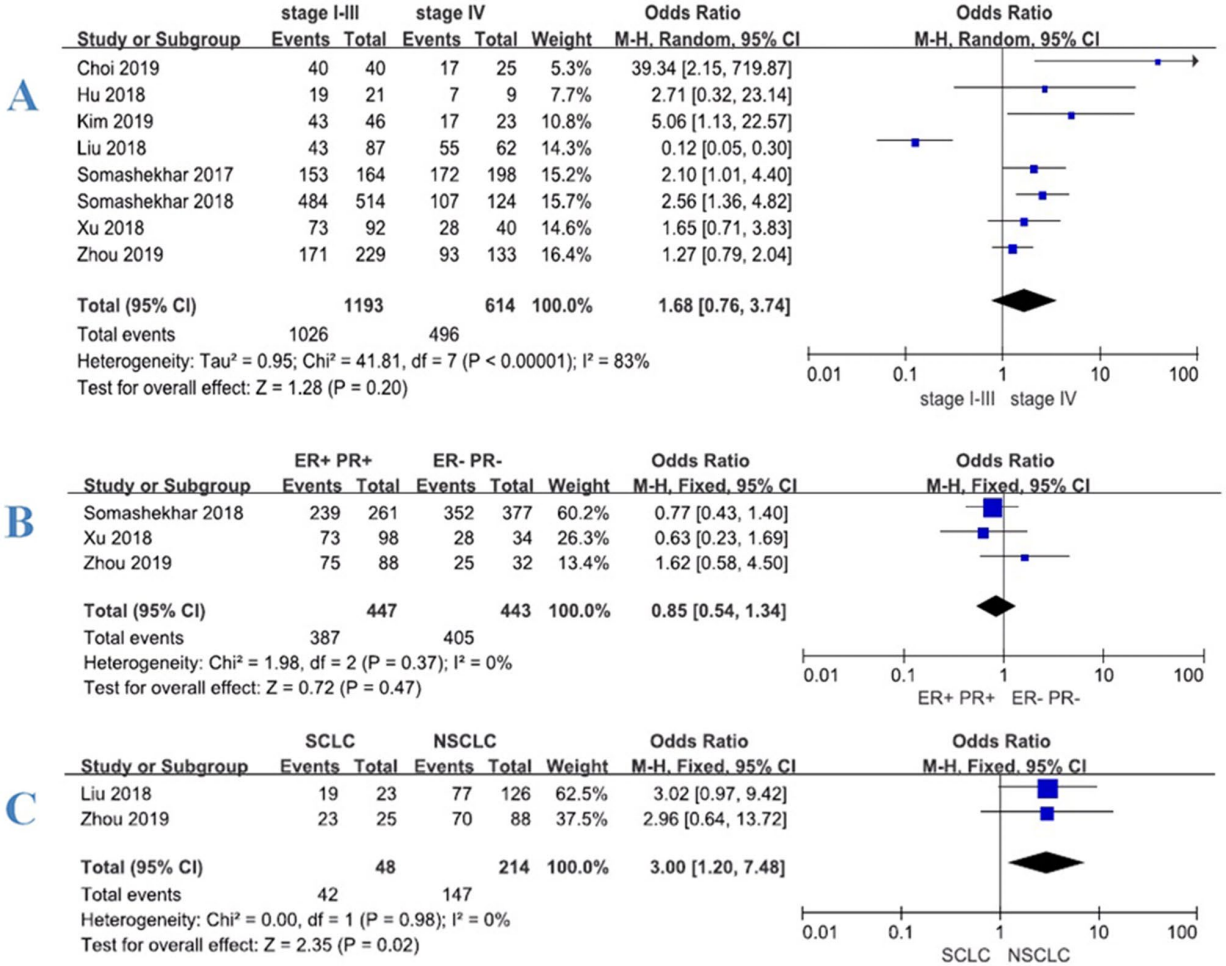
Forest plot of consistency between WFO (‘Recommended’ or ‘For consideration’) and MDT for patients with various cancers. Treatment was considered concordant if the delivered treatment was rated as either ‘Recommended’ or ‘For consideration’ by WFO and discordant if the delivered treatment was either ‘Not recommended’ by WFO or was physician’s choice (not included in WFO). Overall concordance of various cancers in stages I–III and IV (**A**). Concordance of various estrogen and progesterone receptors (ER+/PR+ vs. ER−, PR−) in breast cancer (**B**). Concordance of various pathological types (small cell vs. non-small cell) in lung cancer (**C**).

**Figure 3:**
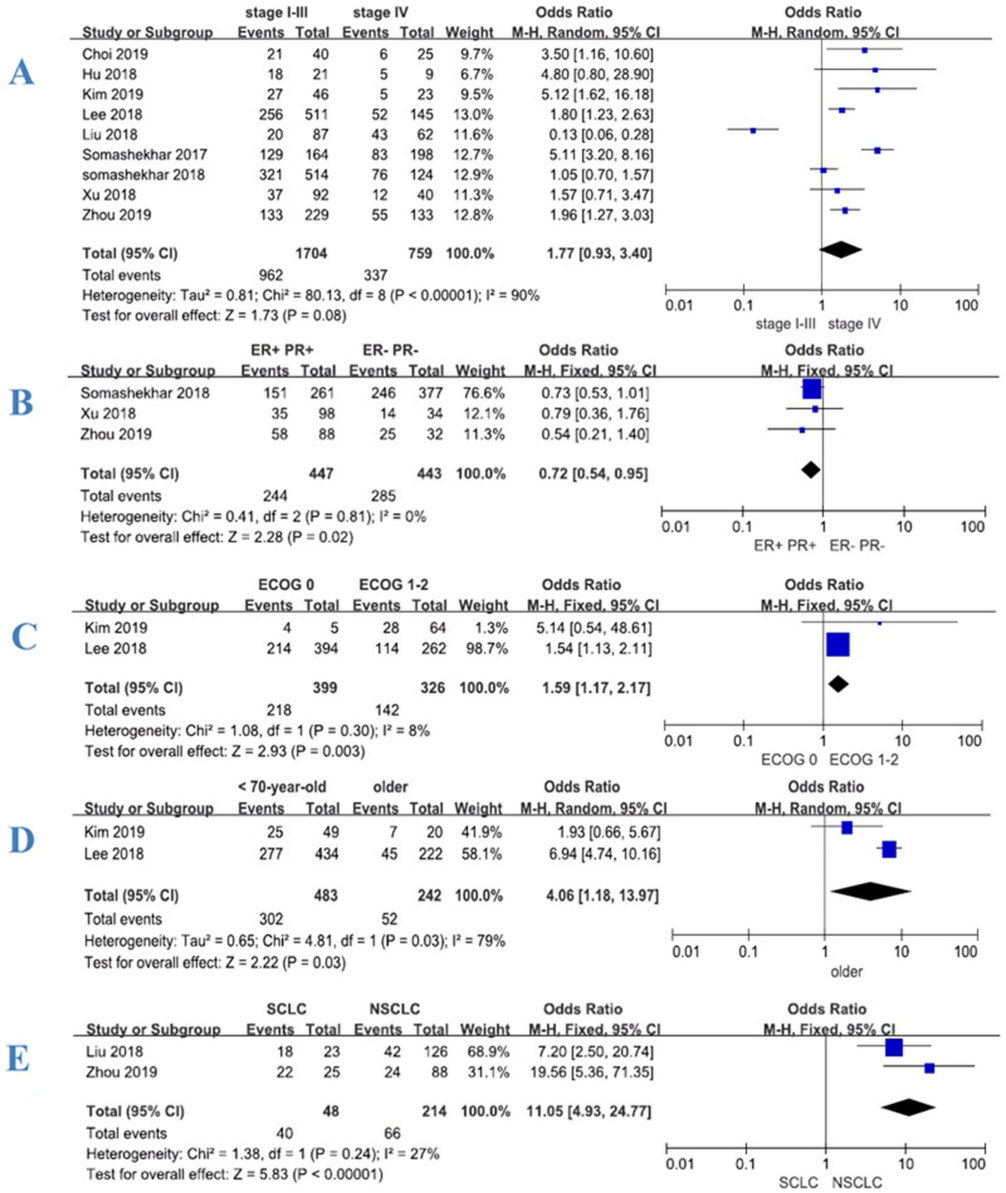
Forest plot of consistency between WFO (only ‘Recommended’) and MDT for patients with various cancers. Treatment was considered concordant if the delivered treatment was rated as ‘Recommended’ by WFO and discordant if the delivered treatment was rated as other options by WFO or was physician’s choice (not included in WFO). Overall concordance of various cancers in stages I–III and IV (**A**). Concordance of various estrogen and progesterone receptors (ER+/PR+vs. ER−, PR−) in breast cancer (**B**). Concordance of various performance status (ECOG 0 vs. ECOG 1–2) in colorectal cancer (**C**). Concordance of various age (< 70-year-old vs. older) in colorectal cancer (**D**). Concordance of various pathological types (small cell vs. non-small cell) in lung cancer (**E**).

Under the condition that the MDT recommendations were consistent with the ‘Recommended’ or ‘For consideration’ categories of WFO, we conducted meta-analysis according to different clinical stages of patients (stage I–III vs. stage IV). A total of 8 studies^15–21,23^ were included in the analysis. Of the 1807 cases included, 1473 (81.52%) WFO treatment schemes were consistent with the MDT. The concordance rate of stage I–III was 86.00% (1026/1193), which was higher than 80.78% (496/614) of stage IV. But the meta-analysis results showed that there was a significant statistical heterogeneity (I^2^ = 83%) at different stages, the meta-analysis was conducted using random effect model (shown in Fig. 2A). The results showed that the difference was not statistically significant, *P* = 0.20 [OR 1.68, 95% CI (0.76, 3.74)]. In order to further analyze the consistency between MDT and WFO, we analyzed the situation that only WFO ‘Recommended’ was included but ‘For consideration’ was excluded. A total of 9 studies^15–23^ were included in the analysis. Of the 2463 cases included, 1299 (52.74%) WFO treatment schemes were consistent with MDT. The consistency of stage I–III was 56.46% (962/1704), which was greater than 44.40% (337/759) of stage IV. The meta-analysis results showed that there was significant statistical heterogeneity (I^2^ = 90%) in different stages (shown in Fig. 3A), so we also conducted the meta-analysis using random effect model. The results also showed that the difference was not statistically significant, *P* = 0.08 [OR 1.77, 95% CI (0.93, 3.40)]. Meta-analysis showed significant statistical heterogeneity (I^2^ > 50%), so subgroup analysis was further adopted according to tumor classification.

#### Subgroup analysis of consistency between WFO and MDT

##### Consistency between WFO (‘Recommended’ or ‘For consideration’) and MDT

**Figure 4:**
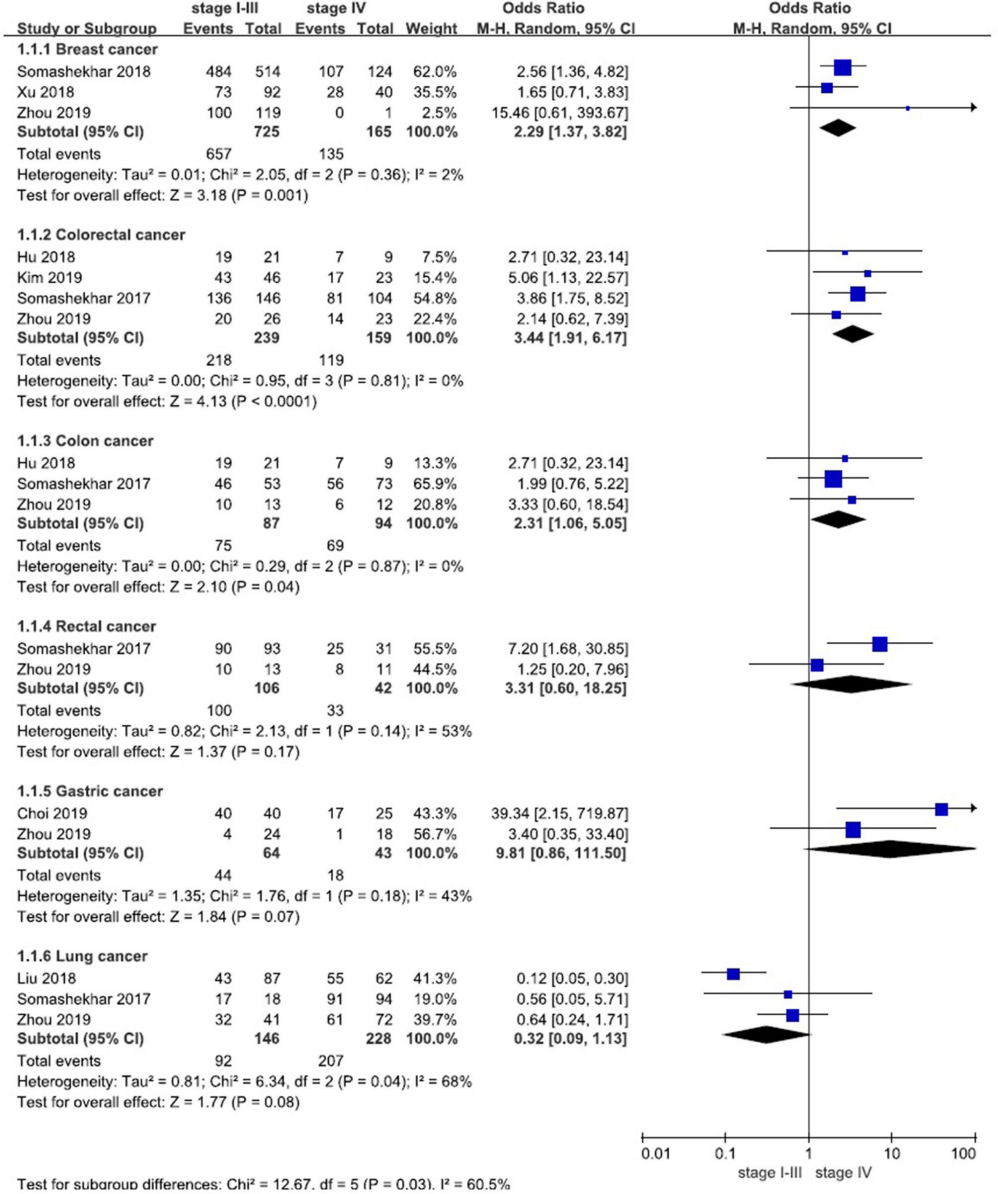
Forest plot of consistency between WFO (‘Recommended’ or ‘For consideration’) and MDT for patients (subgroup).

Under the condition that the MDT recommendations were consistent with the ‘Recommended’ or ‘For consideration’ categories of WFO, we conducted meta-analysis according to different clinical stages of patients (stage I–III vs. stage IV). The results showed that the consistency of stage I–III was greater than that of stage IV except lung cancer (shown in Table 2 and Fig. 4). A total of 3 studies^17,20,21^ (n = 890) were included in our meta-analysis of breast cancer, the results showed that the difference was statistically significant, *P* = 0.001 [OR 2.29, 95% CI (1.37, 3.82)]. A total of 4 studies^16–18,23^ (n = 398) were included in our analysis of colorectal cancer, the results showed that the difference was statistically significant, *P* < 0.0001 [OR 3.44, 95% CI (1.91, 6.17)]. A total of 3 studies^17,18,23^ (n = 181) were included in our analysis of colon cancer, the results showed that the difference was statistically significant, *P* = 0.04 [OR 2.31, 95% CI (1.06, 5.05)]. A total of 2 studies^17,23^ (n = 148) were included in our analysis of rectal cancer, the results showed that the difference was not statistically significant, *P* = 0.17 [OR 3.31, 95% CI (0.60, 18.25)]. A total of 2 studies^15,17^ (n = 107) were included in our analysis of gastric cancer, the results showed that the difference was statistically significant, *P* = 0.07 [OR 9.81, 95% CI (0.86, 111.5)]. A total of 3 studies^17,19,23^ (n = 374) were included in our analysis of lung cancer, the results showed that the difference was not statistically significant, *P* = 0.08 [OR 0.32, 95% CI (0.09, 1.13)].

**Table 2:** Meta-analysis results of consistency between WFO (‘Recommended’ or ‘For consideration’) and MDT for patients with various cancers in stages I–III and IV.

Cancer type	Number of studies	Sample size	Stage I–III		Consistency	Stage IV		Consistency	I^2^	Odds ratio (95% CI)	*P *value
			C	NC		C	NC				
Breast cancer	3	890	657	68	90.62%	135	30	81.82%	2%	2.29 (1.37, 3.82)	0.001
Colorectal cancer	4	398	218	21	91.21%	119	40	74.84%	0%	3.44 (1.91, 6.17)	< 0.0001
Colon cancer	3	181	75	12	86.21%	69	25	73.40%	0%	2.31 (1.06, 5.05)	0.04
Rectal cancer	2	148	100	6	94.34%	33	9	78.57%	53%	3.31 (0.60, 18.25)	0.17
Gastric cancer	2	107	44	20	68.75%	18	25	41.86%	43%	9.81 (0.86, 111.5)	0.07
Lung cancer	3	374	92	54	63.01%	207	21	90.79%	68%	0.32 (0.09, 1.13)	0.08
Total^a^	8	1807	1026	167	86.00%	496	118	80.78%	83%	1.68 (0.76, 3.74)	0.20

*C* Concordance cases, *NC *nonconcordant cases.

^a^The number of rectal cancer and colon cancer, which overlaps with colorectal cancer, has been excluded from the total. In addition, the total includes the number of ovarian cancer and cervical cancer.

In addition, a total of 3 studies^17,20,21^ (n = 890) provided data on estrogen and progesterone receptors (ER+/PR+ vs. ER−, PR−) in breast cancer patients, so meta-analysis was further carried out. The results showed (shown in Fig. 2B) that there was not statistically significant difference, *P* = 0.47 [OR 0.85, 95% CI (0.54, 1.34)]. A total 2 of studies^17,19^ (n = 262) provided data on pathological types (small cell vs. non-small cell) of lung cancer patients. The results showed that the consistency of small cell lung cancer was higher than that of non-small cell lung cancer (shown in Fig. 2C), and the difference was statistically significant, *P* = 0.02 [OR 3, 95% CI (1.20, 7.48)].

##### Consistency between WFO (only ‘Recommended’) and MDT

**Figure 5:**
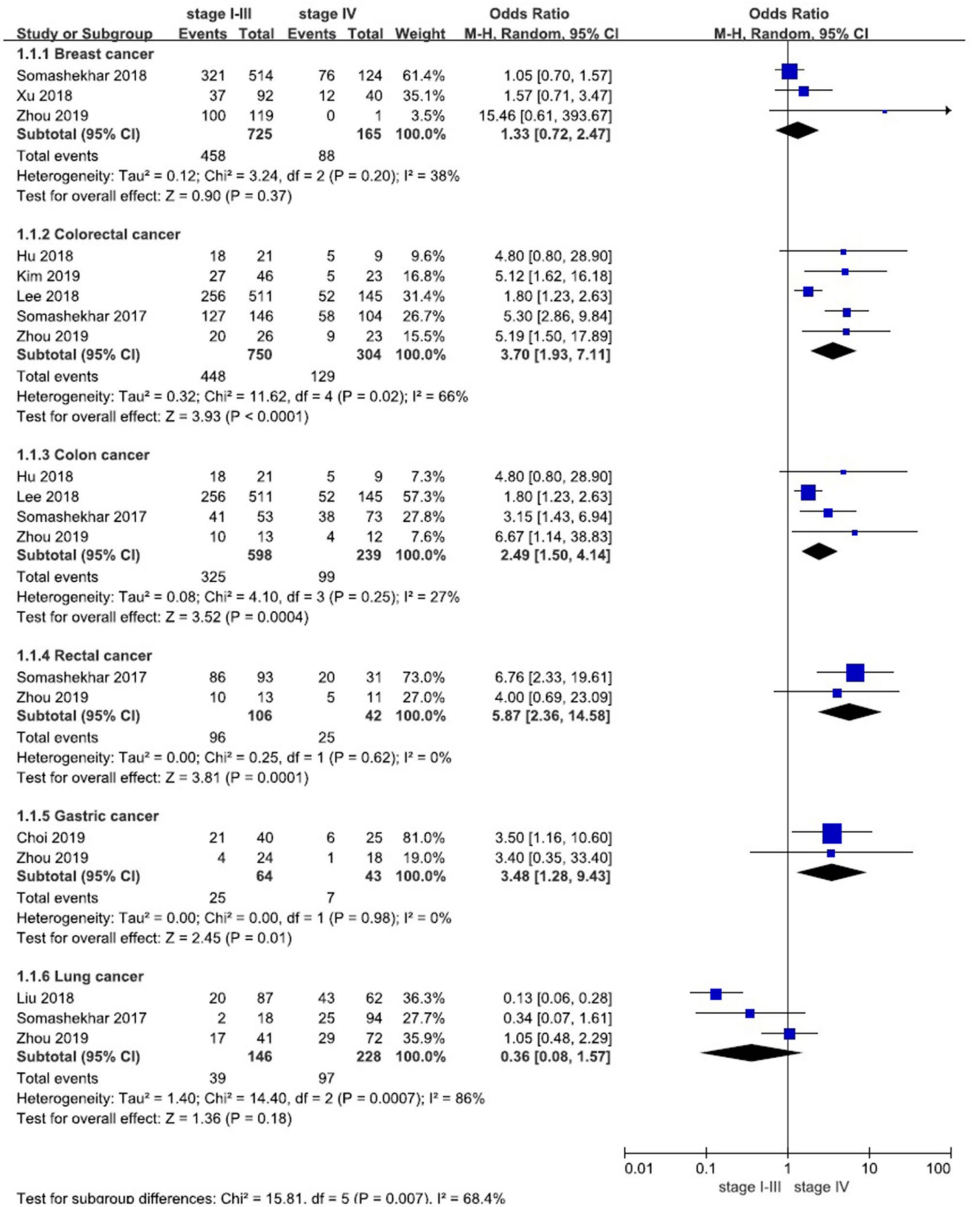
Forest plot of consistency between WFO (only ‘Recommended’) and MDT for patients with various cancers in stages I–III and IV (subgroup).

Under the condition that the MDT recommendations were consistent with only the ‘Recommended’ categories of WFO, we conducted meta-analysis again according to different clinical stages of patients (stage I–III vs. stage IV). Similarly, the results showed that the consistency of stage I–III was greater than that of stage IV except lung cancer (shown in Table 3 and Fig. 5). A total of 3 studies^17,20,21^ (n = 890) were included in our meta-analysis of breast cancer, the results showed that the difference was not statistically significant, *P* = 0.37 [OR 1.33, 95% CI (0.72, 2.47)]. A total of 5 studies^16–18,22,23^ (n = 1054) were included in our analysis of colorectal cancer, the results showed that the difference was statistically significant, *P* < 0.0001 [OR 3.70, 95% CI (1.93, 7.11)]. A total of 4 studies^17,18,22,23^ (n = 837) were included in our analysis of colon cancer, the results showed that the difference was statistically significant, *P* = 0.0004 [OR 2.49, 95% CI (1.50, 4.14)]. A total of 2 studies^17,23^ (n = 148) were included in our analysis of rectal cancer, the results showed that the difference was statistically significant, *P* = 0.0001 [OR 5.87, 95% CI (2.36, 14.58)]. A total of 2 studies^15,17^ (n = 107) were included in our analysis of gastric cancer, the results showed that the difference was statistically significant, *P* = 0.01 [OR 3.48, 95% CI (1.28, 9.43)]. A total of 3 studies^17,19,23^ (n = 374) were included in our analysis of lung cancer, the results showed that the difference was not statistically significant, *P* = 0.18 [OR 0.36, 95% CI (0.08, 1.57)].

**Table 3:** Meta-analysis results of consistency between WFO (only ‘Recommended’) and MDT for patients with various cancers in stages I–III and IV.

Cancer type	Number of studies	Sample size	Stage I–III		Consistency	Stage IV		Consistency	I^2^	Odds ratio (95% CI)	*P *value
			C	NC		C	NC				
Breast cancer	3	890	458	267	63.17%	88	77	53.33%	38%	1.33 (0.72, 2.47)	0.37
Colorectal cancer	5	1054	448	302	59.73%	129	175	42.43%	66%	3.70 (1.93, 7.11)	< 0.0001
Colon cancer	4	837	325	273	54.35%	99	140	41.42%	27%	2.49 (1.50, 4.14)	0.0004
Rectal cancer	2	148	96	10	90.57%	25	17	59.52%	0%	5.87 (2.36, 14.58)	0.0001
Gastric cancer	2	107	25	39	39.06%	7	36	16.28%	0%	3.48 (1.28, 9.43)	0.01
Lung cancer	3	374	39	107	26.71%	97	131	42.54%	86%	0.36 (0.08, 1.57)	0.18
Total^a^	9	2463	962	742	56.46%	337	422	44.40%	90%	1.77 (0.93, 3.40)	0.08

*C *Concordance cases, *NC *Nonconcordant cases.

^a^The number of rectal cancer and colon cancer, which overlaps with colorectal cancer, has been excluded from the total. In addition, the total includes the number of ovarian cancer and cervical cancer.

In addition, a total of 3 studies^17,20,21^ (n = 890) provided data on estrogen and progesterone receptors (ER+/PR+ vs. ER−, PR−) in breast cancer patients. The meta-analysis results showed that the consistency of hormone receptor-positive patients (Luminal A and Luminal B) was lower than that of negative patients (HER2 positive and triple negative), and the difference was statistically significant, *P* = 0.02 [OR 0.72, 95% CI (0.54, 0.95)] (shown in Fig. 3B). A total of 2 studies^16,22^ provided data of different performance status (ECOG 0 vs. ECOG 1–2) and age (< 70-year-old vs. older) of colorectal cancer patients. The results showed that the consistency of ECOG 0 patients was higher than that of ECOG 1–2 patients and the difference was statistically significant, *P* = 0.003 [OR 1.59, 95% CI (1.17, 2.17)] (shown in Fig. 3C); the consistency of patients under 70 years old was higher than that of older, the difference was statistically significant, *P* = 0.03 [OR 4.06, 95% CI (1.18, 13.97)] (shown in Fig. 3D). A total of 2 studies^17,19^ (n = 262) provided data on pathological types (small cell vs. non-small cell) of lung cancer patients. The results also showed that the consistency of small cell lung cancer was higher than that of non-small cell lung cancer, and the difference was statistically significant, *P* < 0.00001 [OR 11.05, 95% CI (4.93, 24.77)] (shown in Fig. 3E).

## Discussion

### Consistency analysis between WFO and MDT

On the whole, it is found that the consistency of stage I–III of other cancers except lung cancer is better than that of stage IV, and most of the results are statistically significant (*P* < 0.05), regardless of setting WFO consistent with MDT at the ‘For consideration’ level (‘Recommended’ or ‘For consideration’) or at the ‘Recommended’ level (only ‘Recommended’). At the ‘For consideration’ level, the overall concordance rate of breast cancer is the highest (88.99%), while that of gastric cancer is the lowest (57.94%). The consistency of small cell lung cancer in patients with lung cancer is higher than that of non-small cell lung cancer, and the difference is statistically significant. At the ‘Recommended’ level, the overall concordance rate of rectal cancer is the highest (81.76%), while that of gastric cancer is still the lowest (29.90%). The consistency of hormone receptor-positive patients (Luminal A and B) of breast cancer is lower than that of hormone receptor-negative patients (HER2 positive and triple negative). In colorectal cancer patients, the consistency of ECOG 0 is higher than that of ECOG 1–2 and under 70 years old is higher than older. However, in lung cancer patients, the consistency of small cell lung cancer is still higher than that of non-small cell lung cancer, and the difference is statistically significant.

### Advantages of WFO

Besides showing high consistency with MDT in most cancers, WFO, as an artificial intelligence clinical decision support system also has the following advantages: (a) WFO improves doctors' work efficiency and reduces workload. Hu’s study^18^ showed that using WFO can save an average of 8.2 min per case (the average time for obtaining reports is 7.3 ± 2.2 min, and the average time for MDT consultation is 15.5 ± 6.1 min). There is no need to wait for MDT to discuss together helps to reduce the time required to formulate chemotherapy scheme^24^, thus shortening the hospitalization time of patients. (b) WFO can prevent man-made calculation errors. Chemotherapy schemes and drug selection involve complicated and time-consuming processes, and there may be errors in selection^25,26^; it can realize accurate medication through computer programs to prevent such errors^20,27^. (c) WFO can improve the quality of doctor-patient communication and prevent doctor-patient disputes. Nowadays, due to a variety of reasons, patients' distrust of doctors is increasing in China^28,29^. The more patients participate in the decision-making of their own therapeutic regimen and understand the incidence of adverse events and other information, the more they have confidence in the therapeutic regimen and will cooperate with doctors more actively^30^. (d) WFO can reduce the burden on patients. It can eliminate the time wasted by patients in consultation in various large hospitals, help patients to obtain the more accurate treatment as soon as possible, avoid fatigue caused by transportation, and reduce travel and accommodation costs while avoiding fatigue caused by travel. (e) WFO can improve the professional level of young doctors. It can significantly shorten the time that junior doctors must spend on consulting relevant documents. At the same time, WFO will give reasons for selection, evidence documents and drug use instructions for each scheme, and update the system once every 1–2 months, thus improving the ability of junior doctors to make accurate diagnosis and treatment recommendations in a short time and improving self-confidence.

### Disadvantages of WFO

Recent studies showed that the consistency between WFO and MDT for cancer patients is not completely consistent, especially in patients with advanced cancer, there is a significant decrease in consistency. It is confirmed that WFO still has certain limitations, which lead to differences in the consistency rate when the system is applied in other countries. The limitations are shown as follows: (a) Different treatment schemes: yellow and white people have significant differences in sensitivity and tolerance to certain specific chemotherapeutic drugs due to their different constitutions and key enzyme groups of drug metabolism, so that clinical guidelines between different countries and regions must also have certain differences. For example, the mutation rate of EGFR in lung cancer in European and American countries is about 15%, while that in China is more than 50%^31,32^. In China, primary research drugs Icotinib and Endostar^33–35^ are used to instead of other first-generation epidermal growth factor receptor-tyrosine kinase inhibitor (EGFR-TKI) and bevacizumab, because studies have shown that they are as effective as EGFR-TKI and bevacizumab in lung cancer patients in China^36,37^. Liu et al.^19^ and others have proposed that if WFO system can provide these two alternative therapeutic regimens in ‘Recommended’ or ‘For consideration’, the overall consistency of lung cancer in China can be increased from 65.8 to 93.2%. Xu et al.^21^ also believe that the difference in first-line treatment of advanced breast cancer can also be attributed to the fact that CDK4/6 inhibitors cannot be used because they are not listed in China. Similarly, WFO recommended panizumab targeted therapy in colon cancer patients, but it is not listed in China and patients cannot choose it^38^. (b) Different drug choices: WFO recommended chemotherapy regimen complies with NCCN guidelines, but it also includes thousands of clinical practice cases from MSK^16^. For example, due to the large difference between the surgical methods and guidelines for adjuvant treatment of gastric cancer in China and the United States^39,40^, the WFO applied research on gastric cancer in the study shows poor concordance rate. On the contrary, the adjuvant therapy and drug selection for colon cancer in eastern and western countries are more consistent, so the concordance rate between WFO and MDT is obviously increased. Liu et al.^19^ also suggested that WFO recommended concurrent chemoradiation during the treatment of lung cancer, whereas China performs sequential chemoradiation (up to 67%). Chinese patients often cannot tolerate concurrent radiotherapy and chemotherapy because their physique is usually weaker than that of western patients. The physique of Chinese patients is usually weaker than that of western patients, which leads to the decrease of coincidence rate between WFO and MDT. (c) Complications: comprehensive treatment for cancer patients is continuous, and patients may suffer from reversible and transient organ function damage. WFO may sometimes exclude some available schemes in the process of selecting the candidate scheme only based on the transient abnormal biochemical results of the patient^41^. In Hu's study^18^, a biochemical blood test of a colon cancer patient showed creatinine clearance rate < 30. WFO did not recommend CapeOX (oxaliplatin + capecitabine) scheme for the patient, but MDT considered that this was only the result of transient biochemical abnormality of the patient, so creatinine clearance rate was rechecked one week later and the result was > 30, so CapeOX scheme treatment was still carried out. In Liu's study^19^, a patient with active pulmonary tuberculosis was also diagnosed as stage III squamous cell lung cancer. If the standard chemoradiotherapy recommended by WFO is accepted, tuberculosis may spread rapidly, resulting in rapid death. Therefore, Liu et al. modified the treatment strategy to oral anti-tuberculosis drugs before radiotherapy and chemotherapy. Therefore, it is believed that if such individualized information can be incorporated into WFO, the coincidence rate between WFO and MDT will be greatly improved. (d) Economic factors: for example, in the treatment of breast cancer, WFO recommends the use of trastuzumab for HER2 positive patients, but patients in China are often forced to choose chemotherapy first due to the high price of this drug^38^. In the Republic of Korea, both WFO and MDT recommend regorafenib for patients with stage IV rectal cancer^42^, but some patients still received 5-fluorouracil (5-Fu)-base chemotherapy, because regorafenib is not only expensive, but also not covered by the national health insurance system^16^. Similarly, China also needs to consider the issue of medical insurance reimbursement, which also affects the consistency between WFO and MDT. If WFO can make targeted improvements to the treatment recommendations for patients with advanced cancer, non-small cell lung cancer, breast cancer with hormone receptor-positive and colorectal cancer with ECOG 1–2 or older (age > 70), it will be more suitable for clinical use in other countries.

### Characteristics and limitations of this meta-analysis

Although WFO has been gradually developed in many countries and regions, and the types of cancers supported are also gradually increasing, so far there is still a lack of evidence-based medicine research for this system. In order to understand the consistency between WFO and MDT, WFO advantages and disadvantages in clinical use, and to solve the practical problems encountered in the practical use of the system, we carried out a targeted meta-analysis. Unlike most of the original studies, which only carry out the consistency research at the ‘For consideration’ level (‘Recommended’ or ‘For consideration’) or at the ‘Recommended’ level (only ‘Recommended’), this research respectively carries out meta-analysis of the above two aspects, which further supports some statistical results obtained from the original studies and provides new statistical evidence. It not only reminds clinicians to pay enough attention to patients with advanced cancer, non-small cell lung cancer, Luminal A and B breast cancer and colorectal cancer with ECOG 1–2 or older (age > 70) in the future when using WFO, but also provides clinical evidence for improvement of WFO. Of course, this meta-analysis still has certain limitations, which are mainly manifested in the following aspects: (a) The possibility of selection bias may exist in a few included studies; (b) The number of samples included in some studies is relatively small, and some study results are not fully reported, lacking complete data of the four classifications. (3) Most studies did not mention the relevant data of WFO's advantages such as shortening consultation time and coincidence between junior or senior doctors and WFO, which leads us to fail to further analyze some of WFO's advantages. (d) All data are published research or conference summaries, lack of grey literature, and possible literature selectivity bias. In addition, 182 cases were included in the initial stage in Liu's study on lung cancer^19^. In the further study, a total of 33 cases were excluded from the study without the support of WFO, and the remaining 149 patients were included in the study. However, the clinical stages of these 33 cases are not listed in detail and cannot be included for further Meta-analysis. Moreover, the distribution of patients in this study is unbalanced, that is, there are fewer patients in early stage, which is obviously different from the situation that there are more early-stage patients than late-stage patients in other cancers. All these may lead to different conclusions about lung cancer from other cancers. Of course, the sample size included in our systematic evaluation is small, so larger sample size, multi-center and high-quality randomized controlled trials are still needed for further verification in order to reach more reliable conclusions.

To sum up, we should regard WFO as "a tool, not a crutch"^43^. If WFO is properly used, it will be regarded as a valuable tool. Proper use requires WFO to be only in the position of a complement to the doctor's work, instead of relying on it completely. Oncologists can integrate it with traditional resources such as colleagues' experience and scientific journals to choose the most effective method to provide chemotherapy schemes for patients, to help patients obtain more accurate and effective treatment, fasten and improve their treatment results. Of course, WFO should also make continuous improvement according to clinical use in other countries. People often say that AI will change medicine. In fact, through examples like WFO, we can look forward to how AI can enable people all over the world to obtain the best quality medical services fairly, no matter where or who the patients are^44^.

## Supplementary Information

Supplementary Figure 1.

Supplementary Figure 2.

Supplementary Figure 3.
