# Supplementary figures and images for: A meta-analysis of Watson for Oncology in clinical application
Source: Sci Rep. 2021 Mar 11;11:5792. doi: 10.1038/s41598-021-84973-5 (PMC7952578; doi:10.1038/s41598-021-84973-5)

**Supplementary
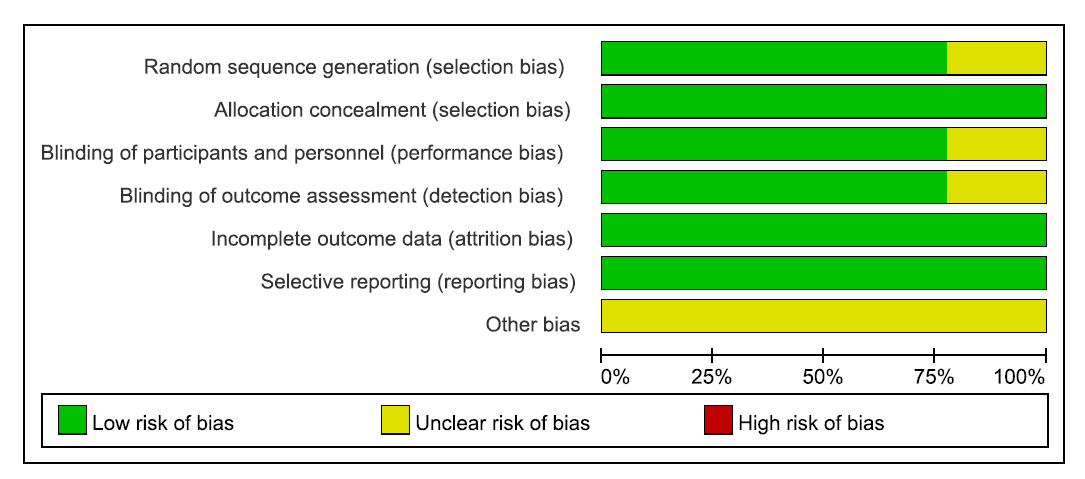
Figure 1.**  Risk of bias graph.

Supplement: Supplementary file 1 — Supplementary Figure 1. [file 41598_2021_84973_MOESM1_ESM.docx]

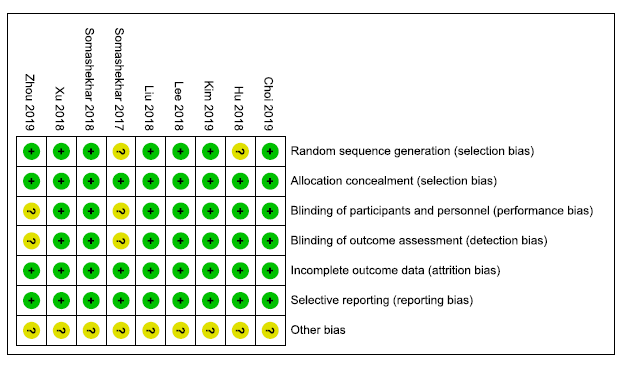


**Supplementary Figure 2.** Risk of bias summary.

Supplement: Supplementary file 2 — Supplementary Figure 2. [file 41598_2021_84973_MOESM2_ESM.docx]
