# Supplementary material for: A meta-analysis of Watson for Oncology in clinical application
Source: Sci Rep. 2021 Mar 11;11:5792. doi: 10.1038/s41598-021-84973-5 (PMC7952578; doi:10.1038/s41598-021-84973-5)
Supplement: Supplementary file 3 — Supplementary Figure 3. [file 41598_2021_84973_MOESM3_ESM.docx]

**Supplementary Figure 3.**  Overall treatment concordance between WFO and the MDT
